# The Use of Plants for Remediation of Metal-Contaminated Soils

**DOI:** 10.1100/tsw.2004.2

**Published:** 2004-01-16

**Authors:** Andon Vassilev, Jean-Paul Schwitzguebél, Theo Thewys, Daniël van der Lelie, Jaco Vangronsveld

**Affiliations:** ^1^ Department of Plant Physiology and Biochemistry, Agricultural University of Plovdiv, 4000 Plovdiv, Bulgaria; ^2^ Laboratory for Environmental Biotechnology, EPFL, CH-1015 Lausanne, Switzerland; ^3^ Centre for Environmental Sciences, Limburgs Universitair Centrum, B-3590 Diepenbeek, Belgium; ^4^ Brookhaven National Laboratory, Biology Department, Upton, NY 11973, USA

**Keywords:** heavy metals, toxic, metals, zinc, cadmium, nickel, lead, copper, mercury, arsenic, metal-contaminated soils, remediation, phytoremediation, phytostabilization, phytoextraction, cost benefit, economical feasibility

## Abstract

The use of green plants to remove, contain, inactivate, or degrade harmful environmental contaminants (generally termed phytoremediation) is an emerging technology. In this paper, an overview is given of existing information concerning the use of plants for the remediation of metal-contaminated soils. Both site decontamination (phytoextraction) and stabilization techniques (phytostabilization) are described. In addition to the plant itself, the use of soil amendments for mobilization (in case of phytoextraction) and immobilization (in case of phytostabilization) is discussed. Also, the economical impacts of changed land-use, eventual valorization of biomass, and cost-benefit aspects of phytoremediation are treated. In spite of the growing public and commercial interest and success, more fundamental research is needed still to better exploit the metabolic diversity of the plants themselves, but also to better understand the complex interactions between metals, soil, plant roots, and micro-organisms (bacteria and mycorrhiza) in the rhizosphere. Further, more demonstration experiments are needed to measure the underlying economics, for publicacceptance and last but not least, to convince policy makers.

## INTRODUCTION

Soil contamination by heavy metals is one of the most serious ecological problems all over the world. Basic sources of this contamination are the metal smelting industry, residues from metalliferous mining, combustion of fossil fuel, and waste incineration, as well as some pesticides and fertilizers used in agriculture, this in addition to soils that are naturally rich in heavy metals. The main metals concerned are cadmium (Cd), lead (Pb), zinc (Zn), copper (Cu), nickel (Ni), mercury (Hg), and the metalloid arsenic (As). When plants accumulate metals, these metals can be ingested by animals, thus creating the potential for toxic effects at higher trophic levels[1]. Actually, metal uptake by plants can play a key role in the entry of metals to terrestrial food chains because vegetation creates habitats for animals and is foraged alive by herbivores or dead by detritivores. Metal toxicity seems to be ascribable to interactions with enzymes (especially those containing SH-groups), structural changes of cell membranes, and phosphorus allocation.

These widespread and persistent environmental pollutants have a high toxicity potential for reproductive and developing tissues and can induce teratogenicity in mammals[2]. Health risks are particularly associated with exposure *in utero* and the early years of life, since the developing organisms are at greater risk from permanent damage, and both absorption and retention can be considerably greater in infants than in adults[3].

It is now well documented that several human diseases or dysfunctions have resulted from chronic exposure to Cd (itai-tai disease[4]), Hg and As (Minamata disease and arsenicosis[5]), and Pb (Pb poisoning[6]). The exposure to acute Cd and Zn concentrations often results in gastrointestinal and respiratory damage as well as damages to heart, brain, and kidney[7]. In addition, soil metal contamination is known to be toxic for animals, micro-organisms, and plants[8,9,10].

The residence time of metals in soil is thousands of years, so they create a permanent risk for human and environmental health and the question of what to do with metal-contaminated soils arises. Of course, the best approach is their remediation, but this is a big task for environmental engineering. The techniques presently used are related mainly to *ex situ* decontamination, which is very expensive and often unacceptable from an ecological standpoint[11]. Therefore, significant research efforts are now addressing other approaches to allow sustainable management of metal-polluted soils.

The choice of a suitable remediation strategy depends on many factors, one of which is the degree of the risk presented by metal-polluted soils. The commonly used physicochemical analyses of soil metal content are not representative enough for risk evaluation, as they do not directly address biological availability and metal toxicity. This prompted the development of biological evaluation tests, including microbial tests for the rapid detection of heavy metal bioavailability[12], which are complementary to the former chemical analysis. In addition, several plant-based tests have been developed for monitoring soil metal phytotoxicity[13,14], but further optimization has also been suggested[15].

Obviously, the heavily contaminated soils from industrial and nonresidential areas should be considered differently from those of agricultural fields, kitchen gardens, and marginally polluted rural areas as they create different risk. The selection of a remediation alternative depends on (1) the size, location, and history of the site; (2) soil characteristics (structure, texture, pH, etc.); (3) the type, physical, and chemical state of the contaminants; (4) the degree of pollution (contaminant concentration and distribution); (5) the desired final land use; (6) the technical and financial means available; and (7) environmental, legal, geographical, and social issues. Previous and projected uses of the soil should be taken into account when considering treatment options.

There are many remediation techniques available for contaminated soils, but relatively few are applicable to soils contaminated with heavy metals. Many soils contaminated with organics can be decontaminated by methods that destroy organics in place. Metals, on the contrary, are immutable and relatively immobile, and so many of the low-cost options available for the remediation of organic contaminants (i.e., thermal volatilization, biodegradation) are not available for metal-contaminated soils. Due to cost, time, and logistical concerns relatively few options remain open.

In general, remediation technologies, whether in place or *ex situ*, do one of two things: they either remove the contaminants from the substratum (“site decontamination or clean-up techniques”), or reduce the risk posed by the contaminants by reducing exposure (“site stabilization techniques”).

One "gentle" plant-based site stabilization approach, suitable for heavily contaminated sites is phytostabilization aimed to decrease soil metal bioavailability using a combination of plants and soil amendments[16]. Although phytostabilization has become more widely accepted, further research is needed concerning the testing of new amendments and the selection of tolerant plant species/genotypes.

Another approach directed towards real decontamination is metal phytoextraction, representing use of plants for metal removal from the soil by concentrating them in the harvestable parts[17]. There is some evidence that metal phytoextraction is a promising approach, but it is still at its infancy stage and needs further development. An opinion exists that phytoextraction will be more economically feasible if, in addition to metal removal, plants produce biomass with an added economical value[18]. There are an increasing number of reports confirming the rationale of this option[19,20,21,22,23].

The present paper aims to summarize available information concerning the development, achievements, and research needs of metal phytoremediation concept, technological, and economical aspects.

## PHYTOREMEDIATION OF METAL-CONTAMINATED SOILS: TECHNOLOGICAL ASPECTS

### Phytostabilization

Plant-based *in situ* stabilization, termed "phytostabilization," thus reduces the risk presented by a contaminated soil by decreasing metal bioavailability using a combination of plants and soil amendments[16,24,25]. This technique is based on the practice of revegetation for the reclamation of mine sites and areas around smelters, in which soil amendments are often essential to establish plant growth. Amendments added to the soil convert the soluble and pre-existing high-soluble solid phase forms to more stable solid phases resulting in a reduced biological availability and plant toxicity of heavy metals. Phytostabilization can also be an interesting alternative for slightly contaminated soils. Due to the application of suitable amendments, metal uptake by (crop) plants, for instance, can be strongly decreased resulting in a reduction of metal transfer to higher trophic levels.

The amendments (for reviews see [15] and [26]) commonly include liming agents, phosphates ($H_3PO_4$, triple calcium phosphate, hydroxyapatite, phosphate rock), metal (Fe/Mn) oxyhydroxides, and organic materials (e.g., sludge or compost). More recent research has investigated other materials that may have value in phytostabilization, including synthetic zeolites, cyclonic and fly ashes, and steel shots.

In phytorestoration, plants perform two principal functions by protecting the contaminated soil from wind and water erosion, and reducing water percolation through the soil to prevent leaching of contaminants[27]. Plants may also help to stabilize contaminants by accumulating and precipitating heavy metals in the roots (or root zone), or by adsorption on root surfaces. Plants may assist in altering the chemical form of the contaminants by changing the soil environment (e.g., pH, redox potential) around plant roots. The micro-organisms (bacteria and mycorrhiza) living in the rhizosphere of these plants also have an important role in these processes: not only can they actively contribute to change the metal speciation, but they can also assist the plant in overcoming phytotoxicity, thus assisting in the revegetation process[28].

Ideally, plants should not accumulate contaminants in above-ground plant tissues which could be consumed by humans or animals and cause harm to these organisms. Phytostabilization of metal-contaminated soils requires metal-tolerant plants and/or plants tolerant to the growing conditions for a given site. Often, the plants chosen for phytostabilization include grasses or other plants that are rather fast growing to provide complete surface coverage, have many shallow roots to stabilize soil and take up soil water, and are easy to care for once established.

Phytostabilization techniques operate like basic farming practices, using similar equipment. The amendments used in phytostabilization may be similar to those used in agriculture (e.g., lime, phosphorus, organic materials). However the application rates required to immobilize metal contaminants are usually much greater than the rates used to fertilize or lime soils. Like any practice that involves plant cultivation, periodical maintenance may be necessary to correct changes in soil fertility, or to correct plant deficiencies or toxicities.

The main objectives for successful *in situ* immobilization are: (1) to change the trace element speciation in the soil aiming to reduce the easily soluble and exchangeable fraction of these elements, (2) to stabilize the vegetation cover and limit trace element uptake by crops, (3) to reduce the direct exposure of soilheterotrophic living organisms, and (4) to enhance biodiversity. The integration of the metal immobilization and subsequent phytostabilization not only results in the installation of a normal functioning ecosystem, but at the same time in an inhibition of lateral wind erosion and reduction of trace element transfer to surface- and groundwater.

It is important to mention that phytostabilization is not a technology for real clean up of contaminated soil, but a management strategy for stabilizing (inactivating) trace elements that are potentially toxic. This should lead to an attenuation of the impact on site and to adjacent ecosystems. Contamination is "inactivated" in place preventing further spreading and transfer into the food chain. Therefore, long-term monitoring of the contaminants will be part of any successful management scheme that uses phytostabilization as a remediation tool.

The basis used for classifying the effectiveness of soil treatment is not standardized and has to be evaluated on a case-to-case basis. For screening the most suitable amendments, different approaches are used in different batch and pot experiments. The effectiveness of the amendments has been assessed in several different ways including chemical methods (e.g., selective or sequential chemical extractions, isotopic dilution techniques, adsorption-desorption isotherms, long-term leaching, and weathering simulations) and biological (e.g., plant growth and dry-matter yield, plant metabolism, ecotoxicological assays on soil invertebrates, and bacterial and microbial populations). One of the lessons learned was the evaluation of the amendments with unpolluted control soils, as some amendments may show undesirable matrix effects (e.g., zeolites with high sodium content destroying soil structure).

The numerous advantages of phytostabilization are very attractive. Effective and durable immobilization of metals reduces their leaching and bioavailability. Subsequently, vegetation can develop to physically stabilize the soil. Over time, a new ecosystem can develop that will increase the biodiversity of the plant species growing. This not only renders the site aesthetically pleasing, but the maturing vegetation cover further provides pollution control and stability to the soil. Lateral wind erosion is completely prevented and metal percolation to the groundwater is highly reduced. The so-called "hard" (or "high-impact") technologies currently available for treating soils contaminated by trace elements are often expensive, destructive, and can generate by-products. Incorporating soil amendments and establishing plant growth are more natural approaches to remediation when compared to some current remediation practices. Compared to "hard" ("high impact") remediation techniques, this technique does not destroy or remove soil organic matter, soil micro-organisms, and soil texture. *In situ* immobilization can be classified as a "soft" ("low impact") site rehabilitation technique. Lastly, *in situ* immobilization can serve as a standby process to reduce the impact of trace element–contaminated soil prior to the use of the most appropriate technologies for clean up.

Through the selection of plants, cropping schemes, and soil amendments, phytostabilization may be adapted to different metal contaminants and soil types, including heavier textured soils, which are sometimes problematic to remediate. This strategy renders the site aesthetically pleasing during and following remediation and helps to restore an (healthy) ecosystem at the site. The vegetation cover further provides pollution control and stability to the soil. Lateral wind erosion is completely prevented and metal percolation to the groundwater is highly reduced. Thus, *in situ* inactivation of metals by strong immobilizing agents combined with subsequent revegetation may be an economically realistic and cost-effective remediation alternative, not only for agricultural soils and kitchen gardens, but also for vast industrial sites, dredged sediment dumps, and other dumping grounds where due to the huge volumes of material to be treated, excavation plus landfilling or cement stabilization are impractical and especially cost inefficient.

Phytostabilization may not be appropriate at certain contaminated sites. Many contaminated sites have less than ideal cultural conditions due to pH, soil structure, salinity, or the presence of other toxic substances. At contaminated sites where soils cannot be made suitable for plant growth without extensive efforts, time, and money, other remediation alternatives should be considered. At sites contaminated with several heavy metals, or a combination of metals and organics, phytostabilization may require an innovative approach. Many heavy metal–tolerant plants are usually tolerant of only one or two metals at high levels, and or not always jointly suited for the remediation of organic compounds. On the other hand, in many cases the degradation of many organic contaminants is strongly stimulated in the plant rhizosphere. In the case of mixed pollution, the selection of the most appropriate vegetation cover should address both metal tolerance and phytodegradation properties. Alternatively, at sites with multiple contaminants, phytostabilization may be conducted in stages with varying crops or treatments, and may possibly be combined with other remediation techniques to provide complete remediation.

## Metal Phytoextraction

Phytoremediation is defined as the use of green plants to remove pollutants from the environment or to render them harmless[29]. Phytoextraction is one of the phytoremediation subareas based on the use of pollutant-accumulating plants for metals and organics removal from soil by concentrating them in the harvestable parts[17]. A recent development of phytoextraction is phytomining — use of plants for accumulation of nickel, thallium, and gold[30].

The idea of using plants to clean up contaminated environment is very old and cannot be traced to any particular source[31], but Chaney[32] was the first who reintroduced it as a remediation concept for growing and harvesting crops on metal-contaminated soils. The concept was initially based on metal hyperaccumulating plants that are able to uptake and tolerate extraordinary levels of metals, much higher than nonaccumulator plants[33,34]. The other approach in the concept's development was based on high biomass–producing plants used together with chemical agents enhancing both metal solubility and uptake by plants[35,36]. In the last decade, extensive research has been conducted to contribute to metal phytoextraction development, searching for new phytoextractors[37,38]; providing more fundamental knowledge about metal uptake, translocation, and plant tolerance[39,40,41,42,43]; as well as improving plant metal accumulation and tolerance by genetic transformations[44]. All these interdisciplinary research efforts have lead to the implementation of the initial concept into promising, cost-effective, and environmentally friendly technologies[17].

In general, a metal phytoextraction protocol consists of the following elements: (1) cultivation of the appropriate plant/crop species on the contaminated site, (2) removal of harvestable metal-enriched biomass from the site, and (3) postharvest treatments (i.e., composting, compacting, thermal treatments) to reduce volume and/or weight of biomass for disposal as a hazardous waste, or for its recycling to reclaim the metals that may have an economic value.

Metal phytoextraction, as any other technology, has both its advantages and limitations. The main advantage of this technology, as often mentioned, is its lower cost as compared to the other known remediation techniques, which is due to the plant's ability to work as a solar-driven pump, extracting and concentrating particular elements from the environment. Direct comparison of the costs associated with landfill excavation and phytoextraction revealed that the cost of the latter is on average ten times lower, calculated from values presented by Glass[45] and the EPA[46]. The economical aspects are discussed more in detail later in this review. The possible metal recycling should provide further economic advantage as the ash of some hyperaccumulators consists of significant amounts of metals (20–40% Zn for *Thlaspi caerulescens*) and there is no need to pay for safe disposal[47]. Another advantage is that phytoextraction can work without further disturbing of the site, which is believed to be of great importance for its public acceptance.

One important limitation of metal phytoextraction is that it can only be used for low to moderately contaminated soils. Very high levels of metal contamination are subjects of other remediation techniques. Another limitation is its applicability only to surface soils (at rooting depth), which varies with the species used, but on average is less than 50 cm. A remarkable exception is the case for some trees, where the target zone is in the range of one to several meters. The application of fast-growing trees, such as *Salix* sp., also offers the possibility to combine heavy metal extraction with the production of biomass for bioenergy production. The options of metal extraction and bioenergy production should, among many other factors, be part of an integrated concept that decides on the feasibility to apply phytoextraction as a remediation technique (see also further in this review: "Economical Aspects").

If cost is the main advantage, time is the greatest disadvantage of metal phytoextraction. It is known that this process is not fast, but (to be realistic for the practical purpose) time should preferably not exceed 10

years or even shorter[31,48]. Another disadvantage is that (as any biological approach) this technology is not capable for full decontamination, because it is limited to the plant-available fraction of the metals. This probably is not a very strong limitation, as contaminated soil has to be cleaned to some degree; for agricultural soils it should be to levels below the threshold value and for industrial or nonresidential soils to the legislative clean-up criteria that can vary per country (see for example Table 1). If remedial action aims at removing only the metal fractions readily available to plants, the time required is also significantly reduced[49]. Hamon and McLauglin[50] referred to this strategy as Bioavailable Metal Stripping.

**TABLE 1**
**Clean-Up Values for Some Metals (mg $kg^{-1}$ Standard Soil) in Residential and Industrial/Nonresidential Areas in U.S. and Belgium[5,31]**

| Metal | U.S. | | Belgium | |
|---|---|---|---|---|
| | Residential | Industrial/ Nonresidential | Residential | Industrial/ Nonresidential |
| Cd | 1 | 100 | 6 | 100 |
| Cu | 600 | 600 | 400 | 600 |
| Zn | 1500 | 1500 | 1000 | 1500 |
| Pb | 400 | 600 | 400 | 600 |
| As | 20 | 20 | 110 | 20 |

Metal phytoextraction technology is still at development stage. Small companies and universities are driving much of its innovation and research, whereas environmental engineering firms are involved in application projects. The available data from finished, full-scale projects are still limited. According to the EPA[51], more data should become available in the next few years. On the other hand, there is evidence that the metal phytoextraction market is continuously increasing. It was evaluated to grow from 15–25 million USD in the year 2000 to 70–100 million USD by the year 2005[45].

At present, there are two basic strategies of metal phytoextraction being developed: *continuous* or *natural* phytoextraction and *induced* or *chemically assisted* phytoextraction[17]. Recently, McGrath et al.[11] introduced the use of fast-growing trees (e.g., *Salix* or *Populus* sp.) as a third option for metal removal. The maximum metal uptake in all these approaches depends on two main variables: metal concentration in harvestable plant parts and biomass yield. Several other facts should also be considered when phytoextraction potential is calculated: the phytoavailable fraction of the metal in the soil, the number of consecutive crops per annum, as well as the metal decrease during the process of extraction.

## *Natural Metal Phytoextraction*

The history of metal hyperaccumulation started at the end of the $19^{th}$ century when it was observed that *Thlaspi caerulescens* (pennycress) and *Viola calaminaria* contained extraordinarily high levels of Zn when growing on soils naturally enriched with this element[17]. This promoted research on the identification of metal hyperaccumulating plants. To date, more than 400 metal-accumulating taxa, belonging to at least 45 plant families, have been identified[34]. They have been found on all continents, both in temperate and tropical environments. Most of the hyperaccumulator plant species are able to accumulate just one metal, but there are also multimetal accumulators. Some populations of *T. caerulescens* are found to have not only high levels of Zn, but also of Cd, Co, and some other metals[52], whereas others do not express this ability[53]. Some families and genera are known as sources of specific metal hyperaccumulators: Ni (*Brassicaceae*:

*Alyssum, Thlaspi*; *Euphorbiaceae*: *Phyllanthus, Leucocroton*), Zn (*Brassicaceae: Thlaspi*), and Cu and Co (*Lamiaceae, Scrophulariaceae*). Several hyperaccumulators are listed in Table 2; for detailed information see Baker and Brooks[37] and Reeves and Baker[34].

**TABLE 2**
**Species Number, Several Metal Hyperaccumulators, and Their Leaf Metal Concentration**

| Metal | Number of Taxa | Several Metal Hyperaccumulators | Metal Content in Leaves (mg $kg^{-1}$ DW) | Ref. |
|---|---|---|---|---|
| Ni | >300 | *Berkheya coddii* | 11,600 | 137 |
| | | *Sebertia acuminata* | 26,000 | 54 |
| Zn | ~30 | *Thlaspi caerulescens* | 39,600 | 138 |
| | | *Minuartia verna* | 11,400 | 139 |
| Cu | ~30 | *Ipomea alpina* | 12,300 | 140 |
| | | *Pandiaka metallorum* | 6270 | 141 |
| Pb | 1 | *Thlaspi rotundifolium ssp. capaefolium* | | 138 |
| Cd | 1 | *Thlaspi caerulescens* | 1800 | 140 |
| As | 1 | *Pteris vittata* | 7000 | 38 |

The term “hyperaccumulation” was introduced by Jaffre[54], describing abnormal levels of plant Ni accumulation, and this term was later extended to the other metals. At present, the criteria used for hyperaccumulation vary per metal and range from 100 mg $kg^{-1}$ dry mass (DM) for Cd, to 1000 mg $kg^{-1}$ DM for Ni, Cu, Co, Cr, and Pb, to 10,000 mg $kg^{-1}$ DM for Zn and Mn. It is pointed out that these values have to be found in any of the above-ground parts of plants growing in their natural habitat, but not under artificial conditions[34]. As usual, these plants exhibit shoot-to-soil metal concentration ratio, the so-called bioaccumulation factor higher than 1[52]. Due to analytical problems, the reliability of some older data has to be considered with care, and to be confirmed, if necessary, by more accurate methods.

Hyperaccumulator plants are usually found on metalliferous soils: calamine soils — enriched in Zn and Pb, serpentine soils — derived from Fe- and Mg-rich ultramafic rocks, enriched also in Ni, Cr, and Co, and other metal rich soils. According to Ernst[55], natural exposure of plants to a surplus of various metals has driven the evolution of metal hyperaccumulation as well as plant resistance to heavy metals. It has been shown that *T. caerulescens* survived for 21 d in hydroponics at 3160 μM Zn without evidence of chlorosis, meanwhile accumulating up to 30,000 mg $kg^{-1}$ DM Zn[56]. Robinson et al.[48] have found Cd accumulation in the leaves of *T. caerulescens* at levels up to 1600 mg Cd $kg^{-1}$ DM without detectable decrease of its dry biomass up to 50 mg extractable Cd $kg^{-1}$ soil. Recently, Lombi et al.[53] found that in hydroponic experiments, one French population of *T. caerulescens* (Ganges ecotype) was able to accumulate Cd in the shoots, over 3000 mg $kg^{-1}$ DM without biomass reduction. Moreover, in field trials, this population was able to accumulate up to 500 mg Cd $kg^{-1}$ DM in the shoot at 12 mg Cd $kg^{-1}$ soil, which is encouraging for the Cd phytoextraction from agricultural soils.

*Pteris vittata* (brake fern) was recently proposed for As decontamination. The levels of As in plants are generally less than 12 mg $kg^{-1}$ DM, but *P. vittata* was found to accumulate As at levels of more than 7000 mg $kg^{-1}$ DM in its fronds, which is hundred times more than any other plant species tested[38]. High capacity for As accumulation was also reported for asparagus fern[57]. The capacity for As accumulation of brake fern together with its ability to cope and survive in many areas with a mild climate as well as its considerable biomass, fast growing, etc. has opened a possibility to be used for As phytoextraction.

Hyperaccumulators are able to survive in their natural environment due to the expression of efficient metal detoxifying mechanisms such as complexation with histidine[40], sequestration in the vacuole[43], binding to phytochelatins (PCs) or metallothioneins (MTs)[39], etc. Detailed information on this subject is also provided by Cobbett and Goldsbrough[58].

In general, the prevailing number of reports assessing metal phytoextraction potential are based on pot experiments, where compared to field experiments higher metal extracting values have been observed: these are due to both higher solubility of metals, the effects of amendments aiming at mobilizing the metals, etc. Recently, some field trial based data have become available[59,60], but as this database is still limited, we also included results from pot experiments in this review.

The first field-based experiment on natural phytoextraction was conducted in 1991–1992 in sewage sludge treated plot at Woburn, England[61]. The greatest Zn uptake was found in *T. caerulescens* accumulating 2000–8000 mg Zn $kg^{-1}$ DM shoots when growing on soil containing total Zn of 150–450 mg $kg^{-1}$. From these data the total Zn uptake was calculated to be 40 kg $ha^{-1}$ in a single growing season. With this extraction rate, it was concluded that it would take nine crops of *T. caerulescens* to reduce total Zn from 440–300 mg $kg^{-1}$ — the threshold value established by the Commission of the European Community[62]. In another trial, supervised by Chaney and collaborators at Pig's Eye landfill site in St. Paul (Minnesota, U.S.), it was found that under optimum growth conditions, *T. caerulescens* could take in Zn at a rate of 125 kg $ha^{-1}$ $year^{-1}$ and Cd at 2 kg $ha^{-1}$ $year^{-1}$[63]. Robinson et al.[48], on the basis of both field observations and pot-soil experiments, concluded that the potential of *T. caerulescens* for Zn and Cd extraction is rather different. They reported Zn removal values very close to that observed by McGrath et al.[61] and suggested that it will be not feasible to remediate the Zn-contaminated mine wastes because of both their high Zn content and low Zn bioaccumulation factor. They considered the case of Cd as different due to very high Cd accumulation in *T. caerulescens* leaves (0.16%) and comparatively lower Cd contamination, especially in some agricultural soils, where phosphate fertilizers have been applied for long periods.

Due to high mobility of Cd in plant-soil system, values exceeding the established food standard (0.1 mg Cd $kg^{-1}$) could appear, for example in grains of durum wheat, sunflower as well as maize, where genotypes with higher Cd accumulation have been observed[64,65]. Thus, there is a need to solve this problem and it seems that it would be entirely feasible by Cd phytoextraction. According to Robinson et al.[48], a single cropping of *T. caerulescens* would reduce 10 mg Cd $kg^{-1}$ soil by nearly a half after 1–2 years only. More realistic data concerning Cd extraction by *T. caerulescens* have been obtained by Schwartz et al.[59] during the work on the EU research project PHYTOREM. The authors measured Cd uptake and mass balance after several years of experimentation on agricultural soil amended with heavy metal–rich urban sludge and found that two crops of *T. caerulescens* extracted about 9% of the total Cd and 7% of the total Zn.

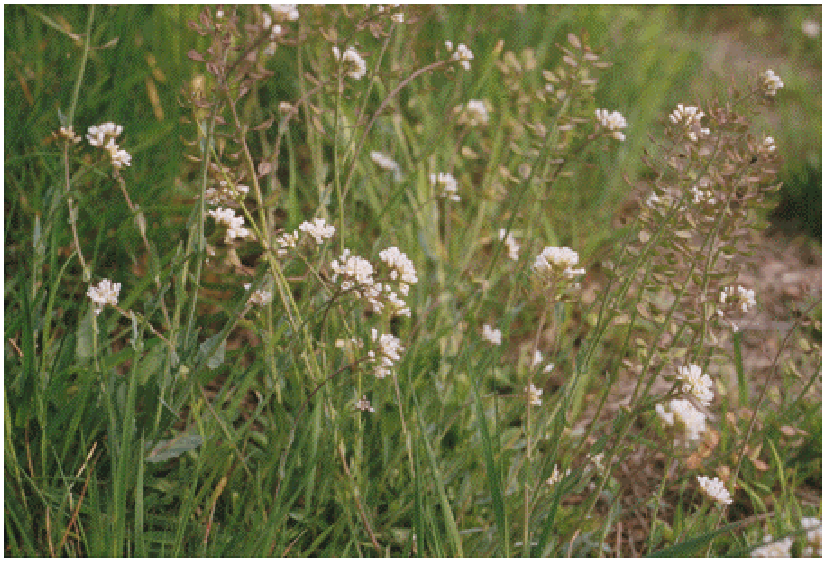

**FIGURE 1.** *Thlaspi caerulescens* (pennycress) on an old zinc/lead mine site in Plombières (Belgium).

If the high metal concentration (Zn, Cd) of *T. caerulescens* is an advantage, its slow growth rate, low dry mass yield, and rosette characteristics are main limitations[66,67]. Field observations and measurements on natural populations of *T. caerulescens* have shown that these plants have an annual biomass production of 2.6 t $ha^{-1}$[48]. Kayser et al.[68] reported a maximum yield from *T. caerulescens* of about 1 t $ha^{-1}$ under field trials due to poor growth and weak resistance to hot environments. On the other hand, Bennett et al.[69] showed that the yield of fertilized crop of *T. caerulescens* could be easily increased by a factor of 2–3 without significant reduction in Zn and Cd tissue concentrations. Recently, Schwartz et al.[59] have shown evidence for this statement observing that Zn and Cd extraction by *T. caerulescens* has been improved significantly by nitrogen fertilization (80–200 mg N kg $soil^{-1}$). Zhao et al.[70] suggested that an average *T. caerulescens* biomass of 5 t $ha^{-1}$ should be achieved with optimized agronomic inputs. Further on, they suggested that it is possible to double this yield in the future by successful screening and plant breeding. Using the target biomass yields (5 and 10 t $ha^{-1}$) and assuming that soil metal contamination occurs only in the active rooting zone (0–20 cm), these authors did some model calculations for Zn and Cd extraction by *T. caerulescens*. For initial concentration of soil Zn of 500 mg $kg^{-1}$, it would take 18–35 crops of *T. caerulescens* to reduce soil Zn to 300 mg $kg^{-1}$ with 10 and 5 t $ha^{-1}$ biomass, respectively. In the case of Cd, 5–9 crops would be required to reduce soil Cd concentration of 5–3 mg $kg^{-1}$. If the aim of the phytoextraction is only to strip bioavailable Cd from soil as has been proposed by Hamon and McLaughlin[50], the time will be much shorter. For example, Schwartz et al.[59] reported that the availability of Cd and Zn (assessed by $NH_4NO_3$-extraction and by growing lettuce as next crop) decreased significantly, more than 70% in the case of Zn. However, the available data showed that even using the best-known hyperaccumulator, *T. caerulescens*, it is not easy to clean heavily contaminated soils and, if possible, it would take a long time. So, there is a need for enhancement of natural phytoextraction potential and several recent studies have addressed this problem[71,72].

The plant-rhizosphere interactions controlling metal uptake by roots are of primary interest. It is necessary to identify which are the main limiting factors and to find appropriate solutions to overcome them. There is some evidence that diffusion of metals is such a limitation as calculations showed that, even in moderately contaminated soils, mass flow contribution is less than 10% of total metal uptake[11]. The diffusion rate in soil generally depends on metal availability in soil solution and, on the other hand, on the concentration gradient driven by metal ions' uptake by roots. It was found that roots of *T. caerulescens* responded positively to Zn and Cd supply[72], but not to enhanced metal solubility by changes in rhizosphere pH[71]. To what extent root exudates can mobilize metals (as was shown for Fe and possibly Zn[73]), or if microbial rhizosphere communities stimulated by these root exudates[74] can contribute to metal phytoavailability, remains to be further examined. As certain plants can use microbial siderophores to improve their Fe uptake, it has been hypothesized that bacterial metal chelators, such as siderophores, can eventually improve the uptake of heavy metals by plants[28,75].

## Chemically Assisted Metal Phytoextraction

Chemically assisted phytoextraction is based on the use of nonaccumulator plants with metal accumulation levels far below those of hyperaccumulators, but with high biomass potential. In general, this approach is aimed to overcome the main limitations of natural phytoextraction — a very limited number of suitable hyperaccumulators for some important metal pollutants such as Pb[76,77], several radionuclides[17] as well as their low biomass.

According to Blaylock and Huang[31], plants that are able to yield more than 20 t $ha^{-1}$ $year^{-1}$ with concentration of the targeted metals of about 1% in the harvestable dry mass should be used for the successful implementation of this phytoextraction technology. The requested dry mass potential is not a limitation as maize, sunflower, and other crops have even higher yields. Thus, the main attention is focused on how to achieve high shoot metal concentrations. It is considered that three key factors control shoot metal accumulation: metal solubility, metal absorption by roots, and metal translocation from roots to shoots.

In general, only a part of total metal content is phytoavailable, mainly the one that is present as free ions, soluble forms, and absorbed to inorganic constituents at ion exchange sites. Some metals such as Zn and Cd occur in exchangeable forms, while others as Pb are less bioavailable and are mainly being precipitated[49]. In any case, to achieve the requested metal uptake value, the concentration of soluble metals in soil must be enhanced. It has been identified that it is possible by rhizosphere manipulation based on the application of chemical agents.

Blaylock et al.[36] and Huang et al.[35] found that application of EDTA (ethylenediamine-tetraacetic acid) at 2 g $kg^{-1}$ soil resulted in a concentration of more than 1.5% Pb in the shoots of Indian mustard (*Brassica juncea*) and about 1% in maize and pea plants. Another chelator HEDTA (hydroxyethyl-ethylenediamine-triacetic acid) applied at the same concentration was found to be able to induce the same level of Pb accumulation in Indian mustard. It was also shown that other chelators such as EGTA (ethylene-bis [oxyethylenetrinitrilo] tetraacetic acid) had high affinity to Cd, while DTPA (diethylene-triamine-pentaacetic acid) showed high affinity to Zn[36]. The application of the chelators at high dosage resulted, however, in severe phytotoxicity: it made plants stop growing and eventually die, reasons why the plants had to be harvested at early growth stages and shortly after treatment with the chelators. On the other hand, Barocsi et al.[60] have recently shown an optimized EDTA application procedure allowing an increase of plant damage threshold, leading to higher metal phytoextraction. Instead of single application, these authors used the chelator in multiple doses, thus monitoring and controlling the EDTA-induced metal accumulation and phytotoxicity, and were so able to achieve maximum removal. Ensley et al.[78] have shown further possibilities to increase metal removal through a combined treatment of EDTA and the nonselective herbicide glyphosate.

If the effect of EDTA on metal solubility in soil can be solely explained by well-established equilibrium principles[79], its influence on plant metal uptake and translocation within plants still is not fully understood. One possible explanation is that it prevents Pb precipitation through forming Pb-EDTA complex, readily available for uptake and translocation, thus probably by-passing the physiological barriers in the roots[80,81,82,83].

Restrictions apply, however, to both the use of complexing agents and artificial soil acidification. It was found that EDTA and EDTA-heavy metal complexes are toxic for some plants and that high doses of EDTA inhibited the development of arbuscular mycorrhiza[82,84,85]. Furthermore, EDTA is poorly photo-, chemo-, and biodegradable[86]. *In situ* application of chelating agents can cause groundwater pollution by uncontrolled metal dissolution and leaching. Some evidence supporting this apprehension has been found[79,85], thus mass balances to confirm that metals are not leached to groundwater have been recommended[18]. Recently, Wenzel et al.[87] used outdoor pot and lysimeter experiments to provide information that supported the presumed risk of EDTA application. They confirmed that EDTA considerably increased metal liability in soil, but also observed enormously increased metal concentrations in the leachates collected below the root zone. Furthermore, they found that the enhanced metal liabilities and leachate concentrations persisted for more than 1 year after harvest. Split application of EDTA was more effective than a single one in order to induce high metal uptake in canola (*Brassica napus* L.), but the achieved shoot metal concentrations were insufficient to obtain reasonable extraction rates that are required to obtain an efficient phytoextraction process.

However, the problems linked to the EDTA application may be overcome by using other chelating agents, such as NTA (nitrilotriacetate). Recently, Kayser et al.[68] showed that this chelator is able to increase the solubility of Zn, Cd, and Cu by a factor of 21-, 58-, and 9-fold, respectively, but plant accumulation was increased only by a factor 2–3. Some organic acids, especially citric acid, have been reported to enhance uranium (U) mobility and subsequently plant uptake[88]. Ebbs et al.[89] found that at pH 5, adding 0.6 g potassium citrate resulted in a 93-fold increase of U solubility. More information about the trends in phytoextraction of radionuclides is provided by Dushenkov[90].

If chelators are needed to solubilize Pb, which is strongly bound to soil organic matter and soil minerals, this effect is also achievable for Cd and Zn in neutral or slightly alkaline soils by lowering the pH. It was shown that similar, although weaker, effects can be induced by applying elemental sulfur or physiologically acid fertilizers, such as $NH_4SO_4$[49,68]. Chaney et al.[91] noted that there might be some negative effects associated with soil acidification. Kayser et al.[68] found that the application of elemental sulfur (S) on carbonate-rich soils is a useful approach creating minimum risk as it is oxidized gradually by sulfur-oxidizing bacteria. Furthermore, we might suppose that sulfur application could improve metal phytoextraction, such as that of Cd, in two ways: (1) by enhanced Cd solubility in the soil, followed by higher plant Cd uptake; and (2) by improved plant S status, allowing an adequate plant defense response to enhanced Cd loading as well as preventing S deficiency onset.

Chemically assisted phytoextraction was primarily developed for Pb decontamination and has been mainly implemented in the U.S.[18,31]. Pb phytoextraction protocol consists of several elements. First, the site's suitability for phytoextraction is evaluated by field observations and treatability studies. The soil samples taken are analyzed to find magnitude and degree of contamination, speciation of metals, as well as to confirm the possibility to decrease metal concentrations to the target clean-up criteria. On the other hand, the capacities of different plant species/cultivars to survive, uptake, and tolerate metals when grown on that soil are tested. Based on the gathered information, as well as on the local climatic conditions, a suitable plant/amendments combination is selected, and the site is prepared for crop cultivation by traditional agronomical means. The amendments are applied at the appropriate time and way taking special care about the possible leaching of metals in groundwater. When the crop reaches the optimum metal content, which is calculated as a value resulting from yield and shoot metal concentration, it is harvested, disposed, and if necessary, the process is repeated. It was reported that following this protocol, a reduction of Pb level from an average of 984–644 mg $kg^{-1}$ in the top soil layer should have been achieved in Boston and New Jersey, U.S.[31]. It was reported that this was achieved in one growing season using three subsequent croppings of Indian mustard.

## *Use of Fast-Growing Tree Species for Metal Phytoextraction*

The ideal plant for metal phytoextraction has to be highly productive in biomass and to uptake and translocate a significant part of the metals of concern to its shoots. Additional favorable traits are fast growth, easy propagation, and a deep root system. Some tree species, mainly willows (*Salix*) and poplars (*Populus*), exhibit these traits and are already used in phytoremediation programs, primarily for rhizofiltration and phytodegradation of organics in contaminated groundwater[92], but also for Cd phytoextraction from lightly polluted agricultural soils[93]. Greger and Landberg[94] demonstrated the rationale of this option in Sweden, namely: (1) willows are currently being grown on about 15,000 ha in the country as a bioenergy source, (2) high Cd accumulators are identified among the *Salix* species (mainly from *S. viminalis*), (3) the ash contains ten times more Cd than the ash from other forest trees, and (4) a method for Cd removal from the ash is available[95].

In fact, *Salix* species are not metal hyperaccumulators, but it was shown that among different clones there are high accumulators of Cd and Zn. Up to 150 clones of different *Salix* species (mainly *S. viminalis*) have been screened for uptake, transport of metals to shoots, and tolerance to Cd, Zn, and Cu[93,96]. Some Cd accumulators were found to contain up to 70 mg $kg^{-1}$ DW in leaves, which is close to Cd hyperaccumulation criteria of 100 mg $kg^{-1}$[94].

If plant resistance to excess metals in the chemically assisted approach is not the limiting factor (as chemical agents are applied shortly before harvest), in the case of tree species this is very important, because plants have to be able to grow continuously on metal-polluted soil. Due to large variation in shoot Cd concentrations (5–70 mg $kg^{-1}$) found in different *Salix* clones, very different calculated values of Cd removal are given in the literature. With mean leaf concentration of 20 mg Cd $kg^{-1}$ and yield of 10 t $ha^{-1}$, Felix[97] calculated that Cd removal rate by *Salix* is 0.222 kg Cd $ha^{-1}$ $year^{-1}$. Greger and Landberg[94] reported that the cultivation of a high-accumulating clone of *S. viminalis* results in 16% removal of total Cd from soil containing 6 mg Cd $kg^{-1}$ soil, which after recalculation gives at least ten times more Cd removal than shown by the previous author. Recently, Klang-Westin and Eriksson[98] estimated the long-term Cd removal by *Salix* using commercial stands grown on different soil types. The net removal of Cd from the plough layer by *Salix* crop varied between 2.6 and 16.5 g Cd $ha^{-1}$ $year^{-1}$ using 8 t $ha^{-1}$ as the highest biomass value in the models. The authors concluded that *Salix* has a high potential for Cd removal as for a long-term perspective (6–7 cutting cycles = 25 years), it would be possible to extract theoretically a maximum of 413 g Cd $ha^{-1}$. Under optimal conditions, the yield of *Salix* can be much higher, up to 30 t $ha^{-1}$, so the resulted Cd phytoextraction would also be higher[99]. On the other hand, the same authors have recently shown that it is possible to further increase Cd removal by poplar using chelating agents like EDTA, NTA, and DTPA. They found that these agents significantly increased the Cd content in leaves for a short time, but could cause necrosis and leaves abscission at rates of 2 g EDTA and 0.5 g NTA $kg^{-1}$ soil, respectively.

### Choice of Suitable Approach and Crop

From the very beginning of the introduction of the metal phytoextraction concept, one key question is still in debate: "What is preferable — to use metal hyperaccumulator plants or to use high biomass–producing crop species?" Chaney et al.[47] considered that metal hyperaccumulation is a more important trait than dry biomass yield. In support to this assumption, they hypothetically calculated Zn removal by hyperaccumulator and high-biomass plants and concluded that in any case the use of hyperaccumulators resulted in higher metal removal. The opposite opinion also exists. For example, Kayser et al.[68] found that the metal removal capacity of *T. caerulescens* was not very different from that of crop species used, this due to poor growth and weak resistance to hot environments, resulting in maximum DM yield of about 1 t $ha^{-1}$. Ebss et al.[100] came to the same conclusion after observing ten times higher Cd concentrations in *T. caerulescens*, but also ten times less biomass production as compared to the crops used.

Obviously, the choice of the phytoextractor depends on the site characteristics: if crops would suffer from toxicity problems, hyperaccumulators, which in general possess a higher metal tolerance, should have an obvious advantage. Another argument that favors hyperaccumulators is the possible reclamation of Zn from Zn-rich biomass, but Ernst[55,66] pointed out that the real recycling of metals from metal-loaded plants has not been proven up to now, and without this operation, the option of hyperaccumulators may be overestimated. Moreover, the Zn price at the world market is actually too low to make "Zn-recycling" from metal-contaminated soil economically feasible.

On the other hand, if high biomass crops are chosen, which one is the most suitable? Obviously, no general answer exists to this question, as there should be different choices for different cases, but several suggestions should be mentioned. If metal contamination is deeper than 20–30 cm, the choice of deep rooting *Salix* will have an obvious advantage; if Cd is the target metal, the choice of maize over sunflower would be preferable as it is known that cereals are semi-resistant, while dicotyledons are more sensitive to this metal[101]. Additionally, an opinion exists that metal phytoextraction would be more economically feasible if, in addition to the plant role in phytoextraction, the used crops produce biomass with an added value (see also further in this review: "Phytoremediation: Cost Recuperation"). For example, energy crops (oilseed and willow), fibers, and fragrance-producing plants could be used to recover these valuable products[18].

A good example of this approach is the so-called adaptable agriculture implemented in some industrial regions in Bulgaria. Near the city of Plovdiv, about 2100 ha of arable lands have been polluted by heavy metals through dust spreading from a nonferrous metal-producing smelter. These soils are carbonate-rich with a high capacity to immobilize heavy metals, but elevated heavy metal levels have been found at many occasions in the produced crops[102]. Thus, it was accepted to use these soils for growing crop species whose final product is not used for human consumption or as forage for animals. The first experiments have been conducted successfully with some aromatic and medicinal plant species, such as peppermint (*Mentha piperita* L.) and lavender (*Lavandula angustifolia* Mill.)[19,20]. They were grown in pots filled by soil, taken at a distance of 0.5, 3, and 6 km from the production plant, which was contaminated by Cd, Pb, Zn, and Cu as well as an uncontaminated control soil. Only for the soil taken at 0.5 km from the production plant, the yields of herbage and essential oil obtained from these crops were up to 17% lower than from the control soil. However, for the other soils similar production yields were found as for the uncontaminated control soil. Furthermore, the oil as final economic product was not contaminated by heavy metals. Finally, Zheljazkov et al.[21] found that peppermint and corn-mint plants removed moderate amounts of heavy metals from the soil by the harvested biomass, thus in long-term perspective, the cultivation of these crops would contribute to the soil remediation.

Recently, Yankov et al.[22] studied the growth, development, yield, and quality of cotton plants, grown in both metal-polluted soil (from the same region) and nonpolluted soil with similar characteristics. The authors did not find any negative influences of heavy metal contamination on any of the above-mentioned properties. They further established that the processing of fiber with boiling water reduced the contents of Cu, Zn, Cd, and Pb to levels found for the plants grown in noncontaminated soil, and concluded that cotton is suitable for growing on metal-polluted soils in this region. In another study, Yankov and Tahsin[23] characterized sunflower's behavior on metal-contaminated soils in the same region. They also found that soil metal contamination did not significantly affected seed yield, but the Cd content in the seed exceeded the admissible concentrations and the seeds had to be used for technical purposes. Both cotton and sunflower crops extracted significant amounts of heavy metals in the harvested biomass. These studies as well as other reports[103,104] showed that crops for fiber or oil production could be used for profitable crop production accompanied by phytoextraction of metal from polluted soils. Some data about the phytoextraction potential of the chosen plant and crop species are given in Table 3.

## *Metal Phytoextraction Optimization, Research Needs, and Perspectives*

The information presented so far has led to the conclusion that metal phytoextraction has remediation potential that can be used for practical aims. However, there is a great need for its improvement. Salt et al.[17] suggested two different strategies: (1) in a short-time perspective, improvement could be achieved by optimization of agronomic practices; and (2) in a long-term view, by the use of genetically modified organisms (GMOs).

**TABLE 3**
**Phytoextraction Potential of Selected Species for Selected Metals**

| Metal | Plant/Crop | Metal Concentrations (mg $kg^{-1}$) | | Ref. | Possible DM Yield (t $ha^{-1}$) | Possible Heavy Metal Removal (kg $ha^{-1}$ $year^{-1}$) |
|---|---|---|---|---|---|---|
| | | Soil | Leaves | | | |
| Cd | *T. caerulescens* | 10 | 1600 | 48 | 2.6–5.2 | 4.16–8.32 |
| | Poplar, willow | 5 | 53 | 99 | 20 | 1.06 |
| Pb | Indian mustard | — | 280 | 68 | 4 | 1.12 |
| | Corn | 2500 | 225 | 35,76 | 10 | 2.25 |
| Zn | *T. caerulescens* | 500 | 10,200 | 48 | 5.2 | 60 |
| | Sunflower | 360–670 | 150 | 68 | 20 | 3 |
| U | Indian mustard | | 1700–5200 | 90 | 5* | 8.5–26* |
| | Red beet | | 200 | 89 | — | — |
| As | Brake fern | 400 | 6805 | 38 | 5* | 34* |
| | Asparagus fern | 1230 | 1130 | 57 | 5* | 5.65* |

Values of possible yields and calculated metal removal marked by * are proposed by the authors.

In the case of natural phytoextraction, the lack of any protocol with respect to cultivation, pest management, and harvesting practices limits more successful implementation, so it has to be developed. On the other hand, screening for suitable ecotypes among known hyperaccumulators as well as a search for new ones should continue. Great differences have been observed among the diverse genotypes of *T. caerulescens* in Zn and Cd tolerance and Cd uptake[53,105]. Barcelo et al.[106] have recently stressed the need of hyperaccumulators that not only exhibit extraordinary levels of metal accumulation in their harvestable biomass, but also develop better survival strategies at different climatic conditions. For example, *T. caerulescens* is not the best candidate in the Mediterranean area because of its sensitivity to heat and drought. Chaney et al.[107] proposed the development of breeding programs for improved cultivars of hyperaccumulators. A partial success from breeding activities has been reported by Brewer et al.[108], who generated somatic hybrids between *T. caerulescens* and *Brassica napus* and recovered high biomass hybrids with superior Zn tolerance.

The chemically assisted phytoextraction also needs technological optimization. It seems that by appropriate mineral nutrition, it could be possible to significantly increase metal removal. Huang et al.[76] achieved a twofold increase in Pb removal by goldenrod plants just by foliar phosphorus application. Other options include screening programs for genotypes with high metal-accumulation potential together with better resistance abilities to excess metals. A greater than tenfold difference in shoot Pb concentration among 50 species/cultivars screened has been observed[76]. Significant differences in shoot Cd accumulation among maize genotypes have also been reported[64]. Recently, well-expressed cultivar-dependent Cd accumulation and resistance has been shown in barley, but it was concluded that Cd phytoextraction capacity of this crop was not sufficient for practical implementation[109]. Since metal absorption in roots is limited by low solubility in soil solution in many cases, it is necessary that the efforts for selection of appropriate rhizosphere manipulation be continued. There is a need to find cheaper, environmentally benign chemical compounds with chelating properties[77] as well as to better understand the role of rhizospheric bacteria in metal solubility, plant uptake, and tolerance[28,110].

Another possibility that should be considered is the use of Plant Growth Promoting Bacteria (PGPB) that stimulate root formation by plants and also produce siderophores. These siderophores can interact with heavy metals, in certain cases reducing their toxicity and increasing their bioavailability and uptake by plants. Endophytic bacteria can be engineered for increased heavy metal sequestration[111,112]. The (combined) activities of these new bacterial strains will be used to enhance heavy metal uptake and translocation by the host plants. These bacterial siderophores can be considered as natural chelators and the bacterial production of which is in tight equilibrium with plant activity**,** thus improving heavy metal uptake and translocation as part of the phytoextraction process. This actually is studied in the frame of an EC project (PHYTAC (QLK3-CT-2001-00429).

Of course, there are also needs to optimize technology elements like plant density per area, number and alternations of appropriate successive crops, time of harvest as well as pest control, irrigation, etc.

GMOs are expected to greatly contribute to metal phytoextraction, but in several parts of Europe and the U.S., there is still reluctance to accept their introduction[113,114]. The most important achievement in that approach is a transgenic yellow poplar (*Liriodendron tulipifera*) expressing bacterial mercuric reductase gene and able to release elemental mercury ten times more than untransformed controls[115]. The research efforts are mainly aimed to increase MT or PC concentrations in plant cells with the hope to improve resistance as well as metal accumulation and translocation pattern in high-biomass producing species. There are several promising examples of successfully transformed plants exhibiting better phytoextraction capacity tested at a laboratory scale. For example, the expression of mammalian MTs in transformed tobacco plants resulted in improved Cd resistance[116]. Transgenic *Brassica juncea* plants overexpressing bacterial glutathione synthetase gene were found to have both higher Cd uptake and enhanced Cd tolerance[42]. Recently Arisi et al.[117] reported that poplars overexpressing bacterial $\gamma$-glutamylcysteine synthetase showed better Cd accumulation, but not improved Cd tolerance. However, the use of GMOs for phytoextraction still remains an open question as its answer strongly depends on public perception. More detailed information about the achievements in GMOs in view of metal phytoextraction is provided by Krämer and Chardonnens[44] and Mejare and Bülow[118].

## PHYTOREMEDIATION OF METAL-CONTAMINATED SOILS: ECONOMICAL ASPECTS

Phytoremediation is often presented as a low-capital-intensive and so low-cost remediation technique especially relevant for diffuse (moderate) pollution in large areas.

Its economical attractiveness is demonstrated by comparing the phytoremediation costs with those of the more traditional techniques like excavating, soil washing, etc. Such comparisons are only meaningful if there is a common remediation target, which means that the *remediation periods* can vastly differ. From this perspective, phytoremediation — because of the longer time period it needs — has a main disadvantage. If a traditional reclamation technique reaches the target much faster than phytoremediation, an economist would think of the earlier regained revenues on the cleaned site as diminishing the higher costs of that traditional technique when compared with the costs of phytoremediation.

### A Cost-Benefit Approach

In deciding which reclamation technique to adopt, one should consider cost and benefit elements over the whole remediation period. Particular attention should go to the most important cost drivers and benefit elements that strike the balance in favor of phytoremediation. As a first step, such decision making can be assisted by the device called "cost-benefit analysis" in which — at least as far as they are measurable — the evolution of costs and benefits over time of phytoremediation can be taken up. In particular, with respect to the benefits, one has to distinguish between the private and the social approach. The social benefits emanating from soil reclamation not only cover the private benefits for the owner or user of the land, but also take account of the decreased negative external effects. A less-polluted site means a less-risky surrounding for humans. Measuring this external benefit in money terms is not evident however. Whether and how much people are willing to pay to avoid the risks arising from land contamination can (for example) be evidenced from housing values in the neighborhood of the polluted site[119]. Such results are not widespread and research is still developing. In the rest of this review all the benefits (and costs) are to be considered from the private point of view.

Assuming a predefined time period for the study (which can be changed as an element of sensitivity analysis), the cost-benefit approach could distinguish the following items:

1. The cost of the phytoremediation action, i.e., capital and operational costs, will be strongly connected with the pollutant-removal performance of the remediation crop, the soil conditions, the difference between the initial and the target level of pollution, etc. All these items will also determine the length of the remediation period.
2. The lost income that the soil is still generating even in its polluted situation.
3. Possibility that some of these costs can be recovered, e.g., the valorization of the biomass.[1]
4. The regained income of the soil after reclamation, determined by its functional use for which the reclamation target is decisive.

These items have to be considered over a predefined study period, covering the remediation period plus the period of prospected regained income from the "cleaned" soil. From the point of view of the owner of the soil, such a period could be, for example, 30–40 years. Discounting the costs and benefits over the study period, one arrives at the "net present value" (NPV) of the phytoremediation alternative.[2]

[1] We remark that the ITRC, in developing a "decision tree for phytoremediation for polluted soils," formulates the question: "Can the plant waste be economically disposed?" Only the "Yes" answer leads to the advice that "phytoremediation has the potential to be effective at the site."[120, p.14]

[2] In one of the rare investigations on the economic viability of phytoextraction, Robinson et al.[121] follow an approach which goes a long away according to the cost-benefit analysis.

Phytoremediation seems particularly applicable in the context of *"land management"* of large areas where the remediation target can be adapted to (1) the ultimate future land use and (2) the intermediate land use in cases where the area is actually a source of agricultural income. In the latter circumstances, the gradual adoption of phytoremediation crops (accumulators) will depend, among other things, on the repercussions on the income of the local farmers. In this context one can use "labor income per hectare per year" as a measurement concept. It means the gross revenue of any (labor) activity on the soil (before and after reclamation) after deduction of capital and operational costs.

The cost-benefit approach could be represented as in Fig. 2. In this figure, the labor income after sanitation is assumed to be larger than before the phytoremediation. It is also assumed that during the remediation period there is a possibility for a positive labor income stemming, for example, from processing the biomass. This income should be considered as net of all costs — that is, of phytoremediation itself (the "system costs") and of all processing costs involved in valorization of the biomass. In Fig. 2 it is assumed that the activities during the reclamation period give a net profit, so that the labor income during reclamation is positive. Of course, if the revenue from valorization is too small (or absent) to compensate for the costs of phytoremediation, the labor income during remediation is negative.

The "lost labor income" during the remediation period is to be considered as the difference between the (abandoned) revenue from the polluted soil diminished with the possible "labor income during reclamation." From the point of view of the user of the soil, the "lost labor income" forms the "(opportunity) cost" of the reclamation.

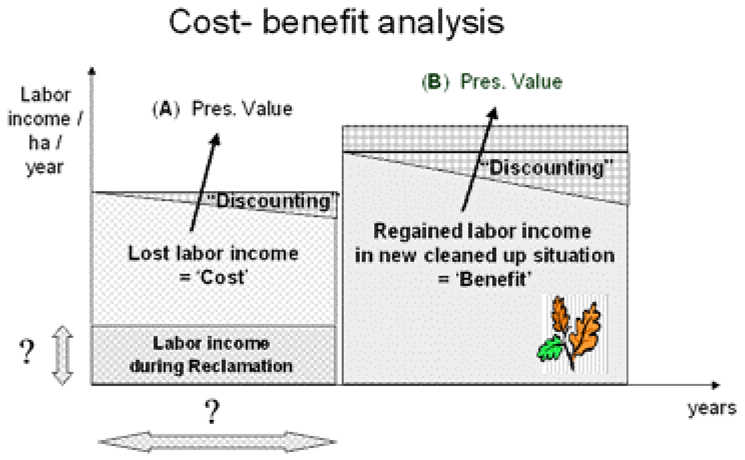

**FIGURE 2**. The framework of cost-benefit analysis.

The NPV is calculated as the difference between the present value of the "regained labor income in the new cleaned-up situation" (B) and the present value of "lost labor income" (A).
This NPV can be used in a number of ways:

1. To analyze its "sensitivity" for changes in important parameters like the distance between the remediation target and the initial level, the removal performance of the crop, the level of regained income versus actual income of the soil, etc.
2. To compare phytoremediation with alternative remediation techniques.
3. To serve as a basis for possible governmental compensation schemes for the lost income during reclamation, in case that the NPV is negative. This compensation can be seen as remuneration for the positive external effects emanating from the cleanup of the polluted site.

## Phytoremediation: Cost Information

Up until now, cost information refers to the costs of the phytoremediation activity (the so-called "system" costs). Measured **absolutely**, these costs vary strongly with specific site conditions, contaminant, crop used, distance to target level, scale of operation, etc. Some examples given by Glass[122] serve as an illustration of this diversity. These figures range per cubic meter from \$1–10/m$^3$ (Cunningham, Dupont), \$29–48/m$^3$ (Salt et al.), to \$100–150/m$^3$ (Chaney, USDA), or per cubic yard from \$10/yd$^3$ (Geragthy & Miller), \$80/yd$^3$ (Levine, DOE), to \$96/yd$^3$ (Jerger et al.), or per ton \$15–20/ton (Drake, Exxon), to \$25–50/ton (Phytotech). For *phytostabilization*, cropping system costs have been estimated at \$200–\$10,000/ha, equivalent to \$0.02–\$1.00/m$^3$ of soil, assuming a 1-m root depth[123]. In the framework of the EU-project concerning the phytoextraction of Pb from soil ("PhyLeS"), the costs involved in the implementation of a *pilot* phytoremediation system amount to approximately 10,000 Euro/100 m$^2$. The latter figure although does not take into account staff costs, since it is assumed that in actual field applications the remediation measures could be carried out either by landowner or by the local environment authority. Being a pilot result, costs could be further curbed in large-scale applications.

This large variability indicates that the ability to develop cost comparisons and to estimate project costs will need to be determined on a *site-specific basis*.

Given this perspective, the economics of phytoremediation are characterized by two considerations: the potential for application and the cost **comparison** to conventional treatments. In making such comparisons, one has to take care to consider the whole system costs that may include[51, p.7]:

1. *Design costs:* site characterization, work plan and report preparation, treatability, and pilot testing.
2. *Installation costs*: (1) site preparation, (2) soil preparation (physical modification: tilling, chelating agents, pH control, drainage), (3) infrastructure (irrigation system, fencing), (4) planting (seeds, plants, labor, protection).
3. *Operating costs*: (1) maintenance (irrigation water, fertilizer, pH control, chelating agent, drainage water disposal, pesticides, fencing/pest control, replanting), (2) monitoring (soil nutrients, soil pH, soil water, plant nutrient status, plant contaminant status).

Given phytoremediation costs being determined on a site-specific basis, a number of authors focused on comparing such costs with more traditional techniques. Bishop[124] states that, at appropriate sites, the cost of applying phytoremediation techniques may range from half to less than 20% of the cost of using physical, chemical, or thermal techniques. Glass[125] and others have estimated that total system costs for some phytoremediation applications will be 50–80% lower than alternatives [51, p.8]. Another overview of such "comparative" results are given by Pivetz[126] who is referring to Blaylock et al., Berti and Cunningham, and Cornish et al. Based on a small-scale field application of Pb phytoextraction, Blaylock et al.[127] mention that the predicted costs for removal of Pb from surface soils using phytoextraction were 50–75% of traditional remedial technology costs. The cost for phytoremediation of 60-cm deep Pb-contaminated soil was estimated by Berti and Cunningham[128] at \$6/m$^2$ (1996 dollars), compared to the range of about \$15/m$^2$ for a soil cap to \$730/m$^2$ for excavation, stabilization, and off-site disposal. Cost estimates made for remediation of a hypothetical case of a 20-in.-thick layer of Cd-, Zn-, and Cs-137-contaminated sediments from a 1.2-acre chemical waste disposal pond indicated that phytoextraction would cost about one-third the amount of soil washing[129].

Robinson et al.[121] used a decision support system based on a cost-benefit analysis over 30 years to assess the viability of using forestry for the remediation of lands contaminated by the 1998 Aznalcóllar mine tailings-dam disaster in Southern Spain. Tree species that could be used for silviculture such as *Pinus pinaster* and *Populus alba* are able to thrive on the contaminated soils. Their calculations indicate that the time needed to phytoextract the heavy metal contamination down to acceptable levels using forestry in 30-year rotations is in the order of hundreds of years. The best alternative technology, the physical removal and storage of the contaminated soil, would take around 2 years. The cost of the soil removal (top 0.3 m) is estimated at USD 500 million for the approximately 4300 ha affected. This equates to USD 116,000/ha. The cost of inaction is estimated at around USD 10,000/ha, largely due to a damaged reputation as a food producer and the potential loss of tourism from the nearby Doñana World Heritage Park. Phytoextraction using forestry for wood production would produce a small profit, currently estimated at USD 2000/ha, every 30 years.

*Conclusion*: When research of phytoremediation began, initial cost estimates predicted that phytoremediation would have lower costs than other remedial technologies. Actual cost data for phytoremediation technologies are sparse, and currently are from pilot-scale or experimental studies that may not reflect accurately expected costs once the technology matures[126]. Cost figures are only meaningful on a site-specific basis, i.e., to make a comparison between the costs of phytoremediation and those of the traditional treatment techniques. In appropriate cases, phytoremediation is looked on as being much more cost effective. The EPA estimates phytoremediation costs to be 50–80% lower than the alternatives for some applications[51]. In most cases, engineering costs are minimal and this, along with the effects of a vegetation cover, helps limit the spread of contamination.

## Phytoremediation: Cost Recuperation

To date, commercial phytoextraction has been constrained by the expectation that site remediation should be achieved in a time comparable to other clean-up technologies. However, if phytoextraction could be combined with a revenue-earning operation, then this *time constraint*, which has often been considered to be the Achilles heel of phytoextraction, may become *less important*. Cost recovery, and the appropriateness of including it as a plant selection criterion, is the subject of increasing current research. The valorization of the biomass has promising avenues especially[121]. As a recent example, Li et al.[130], in developing a technology for commercial phytoextraction of Ni, mention that recovery of energy by biomass burning or pyrolysis could help make phytoextraction more cost effective.

Ways to recover some of the phytoremediation costs could be:

1. The sale of recovered metals when using phytoextraction; however, it might be difficult to find a processor and market for the metal-contaminated plant material[126].
2. Similarly, recovery of costs by selling a commodity type of vegetation, such as alfalfa, lumber, or other wood products, could be difficult due to potential concerns about contaminant residues in the crop. Confirmation that the vegetation is uncontaminated may be required.
3. Valorization of the biomass.

From the outline about the main technologies for biomass conversion[131], we refer to those methods that are relevant for phytoremediation cost recovery:

1. *Biomass direct combustion.* Biomass can be burned in small-scale modern boilers for heating purposes or in larger boilers for the generation of electricity or combined heat and power. Most electricity generation is based on the steam turbine cycle. Biomass combustion systems are in commercial use around the world, using disparate technology. Dedicated combustion plants can burn a wide range of fuel, including wastes. Cocombustion of biomass and coal using pulverized fuel and (circulating) fluidized bed conversion technologies may also be an option.
2. *Biomass gasification.* Biomass gasification converts biomass to a low to medium calorific value gaseous fuel. The fuel can be used to generate heat and electricity by direct firing in engines, turbines, and boilers after suitable clean up. Alternatively, the product gas can be reformed to produce fuels such as methanol and hydrogen, which could then be used in fuel cells or micro turbines, for example. Gasification-based systems may present advantages compared to combustion in terms of economies of scale and clean and efficient operation. Recent gasification activities, in industrialized countries in particular, have focused on fluidized bed systems, including circulating fluidized bed systems. Larger systems coupling combined cycle gas and steam turbines to gasifiers

(biomass integrated gasification combined cycle, BIG/CC) are at the demonstration stage. BIG/CC systems could lead to electrical efficiencies of about 50%.

3. *Biomass pyrolysis.* Biomass pyrolysis produces a liquid fuel that can be transported and stored, and allows for decoupling of the fuel production and energy generation stages. The fuel can be used to generate heat and electricity by combustion in boilers, engines, and turbines. The liquid can also be used to produce a range of specialty and commodity chemicals. Products other than liquid fuels can be obtained from pyrolysis such as charcoal and fuel gas.
4. *Physical-chemical conversion.* The physical-chemical conversion route applies to biomass from which vegetable oil can be obtained, and consists of pressing and extracting oil from the biomass. Vegetable oils can be used in special engines or in diesel engines after an esterification step to produce oil methyl ester. Biofuel from oilseed rape is produced in several European countries.

In an effort to compare these options from the point of view of ecological and economical sustainability — investigated with life cycle analysis — Hanegraaf et al.[132] conclude that the use of crops for electricity is preferred to use of crops for transport fuels since the latter score low on both ecological and socio-economic criteria.

## Energy Production and Land-Take

Treating moderately polluted land with remediation crops that can be used as *energy crops* may be an alternative income for farmers during the remediation period, while at the same time reducing the emission of carbon dioxide. The interest of farmers could also be stimulated when the polluted areas could be treated like set-aside land, where the opportunity exists to grow nonfood crops without losing the existing area grants. Warren et al.[133] demonstrated that gasification of coppice-grown comminuted wood and the subsequent conversion of the gas into electricity are feasible on a farm-sized scale. This work was done on an experimental downdraft gasification plant and engine system capable of producing 30 kW electrical (kWe) and 60 kW of heat.

There is a lot of interest specifically in the use of willow (*Salix* spp.) for phytoremediation. In particular, the use of fast-growing, bushy species, which can be readily grown under a short-rotation coppice (SRC) system, with harvests every 3–5 years, show considerable promise. Burning of the harvested wood to produce renewable bio-energy is also an attractive feature when considering the overall life cycle of the system[134].

It is known that each species of biomass has a specific yield/output, dependent on climate, soil, etc. However, to provide data to concentrate ideas on the involved land-take, it is useful to follow McKendry[135] when he is assuming some general biomass properties. In the case of wood derived from SRC, it is assumed that the average lower heating value (LHV) is 18 MJ/kg. At full generation rate, 1 kg of woodchips converts to 1 kWh(e) via use in a gasifier/gas engine generator, giving an overall efficiency of conversion to electricity of about 20%: this takes no account of the potentially useful heat available from the gasifier/gas engine.

At yields of 15 dry matter ton (dmt) ha-1 year-1 and with 1 dmt equal to 1 MWh(e), 1 ha (based on a 3-year harvesting cycle) of SRC biomass would provide 15 MWh(e)/year. Assuming an annual operating time of 95%, a 100 kW(e) gas engine generator set would require about 55 ha to provide the necessary biomass feedstock, for a 1 MW(e) gas engine generator set, the land-take would be about 550 ha. This calculation suggests that a significant land-take is required to produce a relatively modest energy output as electricity, due to the low overall efficiency of conversion, i.e., 20%, of biomass to electricity. Combustion processes using high-efficiency, multipass, steam turbines to produce electricity can achieve an overall efficiency of 35–40%, reducing the necessary land-take for a 1 MW(e) output to between 270–310 ha. Integrated gasification combined cycle (IGCC) gas turbines can achieve about 60% efficiency. However, the object of McKendry's study was to provide gas to supplement existing landfill gas supplies. Assuming a 20% supplement for a 1-MW(e) landfill gas power generation scheme, the land-take required for SRC is about

110 ha. The author concludes that if biomass with an equivalent calorific value to SRC willow but with a greater crop yield were available, the necessary land-take would reduce in proportion to the increased yield. Reported ranges of yields for *Miscanthus* are quoted as being equivalent to SRC willow at the lower end, while the upper end is about twice that for SRC willow. If this were the case, the land-take for a 20% energy supplement for a 1-MW(e) landfill gas power scheme would reduce from 110 ha for SRC willow to 55 ha for *Miscanthus*. The effect of energy yield in land-take requirements can be seen clearly to be a significant factor in any biomass power generation scheme.

Such results also point to the conclusion that the chances in applying biomass are only realistic in cofiring with traditional energy sources (such as coal) and this in flexible proportions, be it in power stations or in furnaces of ore (e.g., Zn, Cu) smelting plants.

### Environmental Policy

A last observation concerns the role of governmental policy with respect to the viability of biomass as a source for renewable energy. In many cases, power from biomass is not economic because power is generated from a large base of fossil-fueled plants. Mechanisms that will help growers and power plants appropriate a value for the positive externalities of renewable energy are critical to enhance the viability of bioenergy. Hence, one key measure of cost of biomass is the carbon credit (as $/ton $CO_2$ abated) required to equalize the cost of power from a biomass plant with current alternatives. In effect, this is the “premium” associated with the mitigation of green house gasses[136].

## CONCLUSIONS

The information presented in this review describes plants not only as source of food, fuel, and fiber, but also as environmental counterbalances to industrial pollution. In particular, metal phytoextraction has been shown as a promising alternative to the conventional technologies, especially in light to moderately Cd-contaminated soils. However, its wider practical implementation requires further optimizations. As phytoextraction needs a quite interdisciplinary approach, such improvements might be addressed to many plant and soil sciences. Following Clemens et al.[114], it will take quite a while before there will be full understanding of the complex and tightly regulated metal homeostatic network in plants, which is still a major bottleneck in the development of phytoremediation technologies. Further, the improvement of plants by genetic engineering, i.e., by modifying plant metal uptake, transport, accumulation, and tolerance will open new possibilities for phytoremediation.

Phytoremediation, in appropriate situations, is a low-cost technique especially relevant for diffuse (moderate) pollution in large areas. Its main disadvantage is a longer remediation period. That is why, as a decision support tool and to compare with the more traditional reclamation methods, a cost-benefit analysis over a relevant period is appropriate. Possibilities of cost recuperation through the valorization of the biomass can enhance the economic feasibility of phytoremediation. Nevertheless, in many circumstances the NPV resulting from a cost-benefit analysis can be negative. Such a result can serve as a basis for possible governmental compensation schemes. This compensation can be seen as remuneration for the positive external effects emanating from the clean up of the polluted site.

From an economic standpoint, research needs are concentrated on (1) collecting detailed data on costs and benefits from a variety of field experiments; (2) developing “blueprint cost-benefit models” in which the sensitivity of the NPV for important cost drivers (e.g., the pollutant removal performance of the remediation crop, the soil conditions, the difference between the initial and the target level of pollution) of any phytoremediation project can be investigated; (3) the measurement of the private and social benefits of a reclamation, among others in the context of land management. Last but not least, more demonstration projects are required to measure and eventually optimize the underlying economics (feasibility studies) to increase public acceptance and to convince policy makers.

Significant achievements in the mentioned aspects have been obtained during a fruitful coordination of scientific teams in Europe in the frame of COST Action 837 (http://lbewww.epfl.ch/COST837). This collaboration should be continued to further contribute to this emerging and environmentally friendly "green" technology.

## BIOSKETCHES

**Dr. Andon Vassilev** is a plant physiologist at the Agricultural University of Plovdiv, Bulgaria. He received an M.Sc. degree in Agronomy at the Moscow Agricultural Academy, Russia and a Ph.D. degree in Plant Physiology at the Bulgarian Academy of Sciences, Sofia. His studies are focused on both theoretical and applied aspects of plant-metal interactions, mainly on Cd phytotoxicity and Cd phytoextraction. He has worked for 2 years as a postdoctoral researcher at the New University of Lisbon, Portugal and Limburgs Universitair Centrum, Diepenbeek, Belgium.

**Dr Jean-Paul Schwitzguébel** is a plant and microbial biochemist. After his studies at the University of Geneva, he worked as a postdoctoral researcher at the Swiss Federal Institute of Technology (ETH) in Zurich, at the Imperial College of Science and Technology in London, and at the University of Neuchatel, Switzerland focusing on the metabolism and bioenergetics of higher plants and micro-organisms. He moved then to the Swiss Federal Institute of Technology at Lausanne (EPFL) as a permanent senior scientist in the Laboratory for Environmental Biotechnology. Most of his work is now devoted to research and development in the field of plant biotechnology for the removal of organic pollutants from wastewater and contaminated sites. He is also the initiator and coordinator of COST Action 837 on phytoremediation: h

**Prof. Dr. Theo Thewys** is a member of the steering committee of the Centre for Environmental Sciences of the Limburgs Universitair Centrum and head of the Economics & Law Department of the Faculty of Applied Economics. His research is focused on the cost-benefit analysis of soil reclamation in particular, and of relevant environmental regulations in general. First of all the cost efficiency of remediation techniques is investigated. On the other hand, special attention is given to the measurement of the benefits of land reclamation. This includes the private benefits (the regained earning possibilities) and the social benefits (the diminished negative external effects) after remediation. In this respect he conducted studies for several Flemish public environmental authorities.

**Dr. Daniël van der Lelie** is a senior scientist at Brookhaven National Laboratories (BNL), has 13 years experience in environmental biotechnology, and is an expert in the microbiology and ecology of microbial adaptation to pollution by heavy metals and organic xenobiotics. He is strongly involved in the development of technologies for the removal of heavy metals and organics from wastewater and contaminated soils. He is also working on the development and application of biosensors for the detection of bioavailable heavy metals and organic xenobiotics, and has three patent applications in this field. From 1996–2000, he was member of the Editorial Board of the *Journal of Industrial Microbiology and Biotechnology* and since 2001 he is a member of the editorial board of *Biodegradation*. From 1993–1997, he coordinated the EERO network on "molecular adaptation of microorganisms to pollution by heavy metals."

**Prof. Dr. Jaco Vangronsveld** is Director of the Centre for Environmental Sciences of the Limburgs Universitair Centrum. His research is focused on heavy metal uptake by plants, the physiological and molecular effects of metal toxicity, and the transfer of metals to the food chain. Laboratory and *in situ* studies on phytoremediation (phytostabilization and phytoextraction) of heavy metal–contaminated soils started in 1985. Phytoremediation of organics also belongs to the research area of his group, especially on nitroaromatics, oils, and BTEX. Special attention is paid to the interaction between plants and plant-associated micro-organisms (mycorrhiza and bacteria). Jaco Vangronsveld is a member of the Editorial Board of the *International Journal of Phytoremediation* and the *Journal of Soils and Sediments*.
